# Loss of PDZK1 Causes Coronary Artery Occlusion and Myocardial Infarction in Paigen Diet-Fed Apolipoprotein E Deficient Mice

**DOI:** 10.1371/journal.pone.0008103

**Published:** 2009-12-01

**Authors:** Ayce Yesilaltay, Kathleen Daniels, Rinku Pal, Monty Krieger, Olivier Kocher

**Affiliations:** 1 Department of Biology, Massachusetts Institute of Technology, Cambridge, Massachusetts, United States of America; 2 Department of Pathology and Center for Vascular Biology Research, Beth Israel-Deaconess Medical Center and Harvard Medical School, Boston, Massachusetts, United States of America; Harvard Medical School, United States of America

## Abstract

**Background:**

PDZK1 is a four PDZ-domain containing protein that binds to the carboxy terminus of the HDL receptor, scavenger receptor class B type I (SR-BI), and regulates its expression, localization and function in a tissue-specific manner. PDZK1 knockout (KO) mice are characterized by a marked reduction of SR-BI protein expression (∼95%) in the liver (lesser or no reduction in other organs) with a concomitant 1.7 fold increase in plasma cholesterol. PDZK1 has been shown to be atheroprotective using the high fat/high cholesterol (‘Western’) diet-fed murine apolipoprotein E (apoE) KO model of atherosclerosis, presumably because of its role in promoting reverse cholesterol transport via SR-BI.

**Principal Findings:**

Here, we have examined the effects of PDZK1 deficiency in apoE KO mice fed with the atherogenic ‘Paigen’ diet for three months. Relative to apoE KO, PDZK1/apoE double KO (dKO) mice showed increased plasma lipids (33% increase in total cholesterol; 49 % increase in unesterified cholesterol; and 36% increase in phospholipids) and a 26% increase in aortic root lesions. Compared to apoE KO, dKO mice exhibited substantial occlusive coronary artery disease: 375% increase in severe occlusions. Myocardial infarctions, not observed in apoE KO mice (although occasional minimal fibrosis was noted), were seen in 7 of 8 dKO mice, resulting in 12 times greater area of fibrosis in dKO cardiac muscle.

**Conclusions:**

These results show that Paigen-diet fed PDZK1/apoE dKO mice represent a new animal model useful for studying coronary heart disease and suggest that PDZK1 may represent a valuable target for therapeutic intervention.

## Introduction

Hypercholesterolemia is recognized as one of the most important predisposing risk factors for the development of occlusive coronary arterial atherosclerosis and myocardial infarction [1]. Under typical experimental conditions, neither LDL receptor nor apoE KO mice exhibit robust occlusive coronary artery disease associated with myocardial infarction, heart dysfunction and death during the first six months of life (see for example [2]). After 8 months (chow diet) [3] or 5 months (Western diet) [4] of age, apoE KO mice develop atherosclerotic lesions in the proximal segments of coronary arteries, resulting presumably from the extension of lesions present in the aortic root [4]. Occasional myocardial fibrosis has been observed in 10 month old apoE KO mice [4]. In contrast, there is robust aortic root and aortic atherosclerosis in these mice, and they are commonly used as a model of human atherosclerosis [5], [6], [7].

High density lipoprotein (HDL) and its receptor, scavenger receptor class B type I (SR-BI), have been described as atheroprotective [8], [9], [10], [11], [12]. They participate in the transport of cholesterol from peripheral tissues (e.g. atheromatous plaques) to the liver and subsequent excretion into the bile, a process called reverse cholesterol transport [13], [14], [15]. SR-BI is a 509 amino acid membrane-associated protein predominantly expressed in the liver and steroidogenic organs, and also in enterocytes in the small intestine, macrophages and endothelial cells [15], [16], [17], [18], [19], [20], [21].

Several experimental models have shown that overexpression of SR-BI in murine liver decreases the extent of atherosclerosis, even though it decreases HDL-plasma cholesterol concentration [22], [23], [24], [25]. Partial or total loss of SR-BI increases atherosclerosis in several murine models [12], [26], [27], [28].

Mice deficient in both SR-BI and apolipoprotein E (SR-BI/apoE double knockout (dKO) mice) fed a normal chow diet not only exhibit dramatically enhanced hypercholesterolemia and accelerated aortic root atherosclerosis [12], but also exhibit rapid onset occlusive coronary artery atherosclerosis, myocardial infarction and premature death (mean age of death ∼6 weeks of age) [11]. Thus, SR-BI/apoE dKO mice provide a very rapid, small animal model that mimics many cardinal features of human coronary heart disease [2], [11], [29].

PDZK1 is a four PDZ domain protein that binds to and regulates the expression of SR-BI in a tissue specific manner [30], [31], [32], [33]. Loss of PDZK1 in PDZK1 KO mice is accompanied by an ∼95% reduction in hepatic SR-BI levels and a concomitant ∼1.7 fold increase in plasma total cholesterol levels. However, loss of PDZK1 does not affect SR-BI expression in steroidogenic tissues [32] or macrophages [21]. These findings led to the conclusion that PDZK1 is a tissue specific adaptor protein for SR-BI, and joins ARH (autosomal recessive hypercholesterolemia gene) in a new class of tissue specific adaptor proteins for lipoprotein receptors. ARH is an adaptor for the LDL receptor [34] that regulates this receptor in a tissue specific fashion.

We have previously shown that PDZK1 is atheroprotective in mice [21]. PDZK1/apoE dKO mice fed with a high fat/high cholesterol diet (Western diet) develop increased aortic root atherosclerosis compared to apoE single KO mice, but fail to develop occlusive coronary artery disease and myocardial infarction [21].

In this report, we examined the effects of 3 months of feeding another atherogenic diet, the high fat, high cholesterol, cholate containing ‘Paigen’ diet on PDZK1/apoE dKO mice and control apoE single KO mice. We found that the Paigen diet induced a more severe hypercholesterolemia and greater aortic atherosclerosis in PDZK1/apoE dKO mice than in the apoE KO controls. Strikingly, in contrast to the Western diet, the Paigen diet induced development of occlusive coronary arterial atherosclerosis and myocardial infarction in PDZK1/apoE dKO mice not seen in apoE KO controls. Thus, the Paigen diet-fed PDZK1/apoE dKO mice represent a new murine model of coronary heart disease and suggest that PDZK1 may represent a valuable target for therapeutic intervention.

## Materials and Methods

Animal protocols were reviewed and approved by the respective Animal Care & Use Committees at the Beth Israel Deaconess Medical Center and the Massachusetts Institute of Technology.

### Animals

ApoE deficient mice (C57BL/6 background) were purchased from Jackson Laboratories (Bar Harbor, ME), mated with PDZK1 deficient mice (129 SvEv background), and backcrossed for 6 generations into C57BL/6 background to generate the PDZK1/apoE double knockout (dKO) and apoE single KO mice as previously described [21]. As a result, experiments were conducted using apoE single KO and PDZK1/apoE dKO male mice with ∼98.5% C57BL/6/1.5% 129 SvEv background. Genotypes were determined by PCR using established protocols ([35] and Jackson Laboratories web site). For analyses of lipoproteins (n = 8–25 as shown in Table 1) and atherosclerosis (n = 8 per group) and cardiac morphology (n = 8 per group), approximately 4-week-old animals were fed a “Paigen diet” from Harlan Teklad (Madison, WI) containing 7.5% cocoa butter, 15.8% fat, 1.25% cholesterol, 0.5% sodium cholate for 3 months [36].

**Table 1:** Plasma lipid levels and body weights of apoE KO and PDZK1/apoE dKO mice.

Genotype	TC	UC	UC:TC	PL	TG	Wt
apoE KO	1898±62**^a^**	517±14**^b^**	0.27±0.004**^c^**	553±23^d^	67±8	25±0.6**^e^**
PDZK1/apoE dKO	2529±82	770±33	0.31±0.01	751±22	85±9	28±0.5

Four month old animals were fasted for 4 hours prior to sample collection. Values are represented as mean±standard error from 8–25 animals (mean 20 animals) per group. Statistical significance was determined by pairwise comparisons of each value from PDZK1/apoE dKO mice with apoE KO controls by using unpaired Student's t test. The abbreviations and units used are TC (total plasma cholesterol, mg/dL), UC (plasma unesterified cholesterol, mg/dL), UC:TC (plasma unesterified cholesterol to total plasma cholesterol ratio), PL (plasma phospholipids, mg/dL), TG (plasma triglycerides, mg/dL) and Wt (body weights, g).

a, b, c, e P<0.01

d P<0.05

### Morphologic and Biochemical Analyses

Mice were anesthetized. Blood was obtained by cardiac puncture using heparinized syringes and centrifuged to obtain plasma. Hearts were excised after a short *in vivo* perfusion with PBS, weighted and frozen in OCT compound. Transverse frozen sections (10 µm) were stained with Oil Red O/hematoxylin and atherosclerotic lesions were measured by planimetry as the sum of the cross-sectional areas using image measure/SPOT software (Diagnostics Instruments, Sterling Heights, MI) in the aortic root as previously described [12]. Coronary arteries were visually scored according to the severity of their occlusion status as follows: severely occluded (50–100%), partially occluded (10–50%) and minor occlusions (0–10%) [37], [38]. Cardiac fibrosis was evaluated on cryosections stained with Mason's Trichrome (Sigma, St Louis, MO). Digital images of sections collected using a Nikon E600 microscope with a SPOT digital camera and software (Diagnostic Instruments, Inc.) were analyzed using OpenLab software (Improvision, MA) and color-based thresholding segmentation of the images (32 bits/pixel, RGB). To calculate the percentage of cardiac fibrosis, we defined total ventricular myocardial tissue area as the total number of all blue and red pixels in the image, excluding atrial tissue and all valve leaflets. Fibrotic area was then defined as the total number of blue pixels. % cardiac fibrosis  =  (fibrotic area/total tissue area) ×100 [37], [39].

Immunoblots were performed as previously described [40]. Briefly, total liver samples (40–50 µg of protein/sample) were size-fractionated by 10% SDS-PAGE and immunoblotted on nitrocellulose membranes with either polyclonal antipeptide antibodies for SR-BI [16] or for actin (used as protein loading control, Sigma). Antibody binding to protein samples was visualized by enhanced chemiluminescence using Super Signal West Pico Luminal reagents (Pierce, Rockford, IL). In order to determine if the Paigen diet induced changes in the expression of hepatic SR-BI, the relative amounts of SR-BI were measured using a Kodak Image Station 440 CF and Kodak 1D software in liver samples from apoE KO and PDZK1/apoE dKO mice fed chow or Paigen diet for three months (three mice per group). The results were normalized with respect to the level of actin expression in the respective tissue samples.

Immunoperoxidase studies to determine the respective contribution of macrophages and smooth muscle cells in aortic root atherosclerotic lesions were performed using CD68 (Serotec) and alpha smooth muscle actin antibodies (Thermo Scientific) according to manufacturer's recommendation. Five-µm fixed-frozen sections were stained with the anti-CD68 or anti-smooth muscle actin antibodies, followed by biotinylated either anti-rat or anti-mouse IgG, visualized by immunoperoxidase staining, and counterstained with Harris modified hematoxylin, as described previously [32].

Total and unesterified cholesterol, phospholipids and triglycerides were measured using kits (Wako Chemical, Richmond, VA). FPLC size fractionation of plasma lipoproteins was performed as previously described [41].

### Statistical Analyses

A value of P<0.05 between experimental groups was considered to represent a significant difference using a 2-tailed, unpaired Student's *t* test or one-way ANOVA with Tukey's multiple comparisons post-test where appropriate. Reported values represent mean ± standard error of the mean.

## Results

We have previously shown that PDZK1 confers protection against aortic root atherosclerosis in apoE KO mice fed an atherogenic, Western-type, high fat/high cholesterol diet for 3 months [21]. Under these conditions, there was no evident occlusive coronary arterial atherosclerosis nor cardiac damage. To explore further the consequences of PDZK1 deficiency in apoE KO mice on atherosclerosis and the heart, we subjected mice to a more severe, cholate-containing atherogenic diet (Paigen diet). Four week-old apoE single and PDZK1/apoE dKO mice were fed a Paigen diet (7.5% cocoa butter, 15.8% fat, 1.25% cholesterol, 0.5% sodium cholate) for three months. At this stage, plasma was obtained and mice were weighed and sacrificed to analyze the extent of aortic root atherosclerosis and cardiac damage.

**Figure 1:**
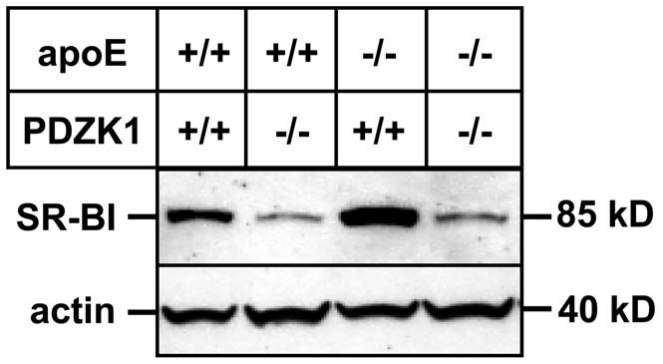
Immunoblot analysis of hepatic SR-BI expression. Mice with the indicated genotypes were fed a high fat/high cholesterol/cholate-containing “Paigen” diet for three months. Livers were harvested and subjected to immunoblotting using anti-SR-BI and anti-actin (loading control) antibodies as described in Materials and Methods.

We previously showed that loss of PDZK1 causes a dramatic reduction in hepatic SR-BI expression in otherwise wild-type mice [32] or in apoE KO mice fed either a standard lab chow or a Western-type diet [21]. However, the effect of PDZK1 ablation on SR-BI levels in the liver of Paigen diet-fed animals was not known. Western blot analyses of Paigen diet-fed animals (Fig. 1) showed a dramatic decrease in the levels of SR-BI protein expression in the livers of PDZK1/apoE dKO animals when compared to apoE KO animals. Quantification experiments showed that the Paigen diet did not change the expression of SR-BI in both apoE KO mice (Relative fold change in SR-BI liver expression in apoE KO mice fed Paigen diet versus chow diet  = 1.20±0.18, p = 0.34) and PDZK1/apoE dKO mice (Relative fold change in SR-BI liver expression in PDZK1/apoE dKO fed Paigen diet versus chow diet  = 1.17±0.60, p = 0.79).

The dramatic reduction in hepatic SR-BI by ablation of the PDZK1 gene in Paigen diet-fed apoE KO mice was accompanied by increased plasma levels of total cholesterol (33%), unesterified cholesterol (49%), and phospholipids (36%) as well as an increase in the unesterified to total cholesterol ratio (14%) and total body weight (13%) (Table 1).

**Figure 2:**
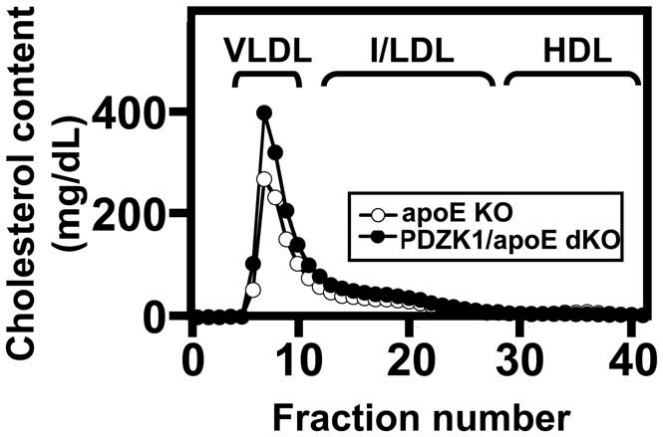
Lipoprotein cholesterol profiles from apoE KO and PDZK1/apoE dKO mice. Plasma harvested from individual mice fed a Paigen diet for three months was size fractionated using FPLC, and the total cholesterol contents of the fractions (mg/dL plasma) were determined by enzymatic assay. Profiles averaged from 2 independent experiments for each genotype, each composed of pooled plasma from six apoE KO (open circles) and six PDZK1/apoE dKO (filled circles) mice per experiment are shown. Approximate elution positions of human VLDL, IDL/LDL and HDL are indicated.

Figure 2 shows the size distribution profiles of cholesterol in plasma lipoproteins measured by FPLC. As previously reported [3], [5], [6], most of the plasma cholesterol in apoE KO mice is carried in VLDL-size particles. The plasma lipoprotein profile of PDZK1/apoE dKO mice was similar, but the VLDL-size peak was higher (Fig. 2). Under the conditions used for these experiments (substantial dilution of the samples because of the high total cholesterol levels), we did not detect HDL-size peaks in any of the samples from PDZK1/apoE dKO mice, whereas we occasionally observed a small HDL peak in apoE KO samples. The unesterified to total cholesterol (UC:TC) ratio of FPLC fractions from the VLDL region (not shown) indicated that the UC:TC ratio was higher in PDZK1/apoE dKO mice, consistent with the findings from total plasma measurements.

**Figure 3:**
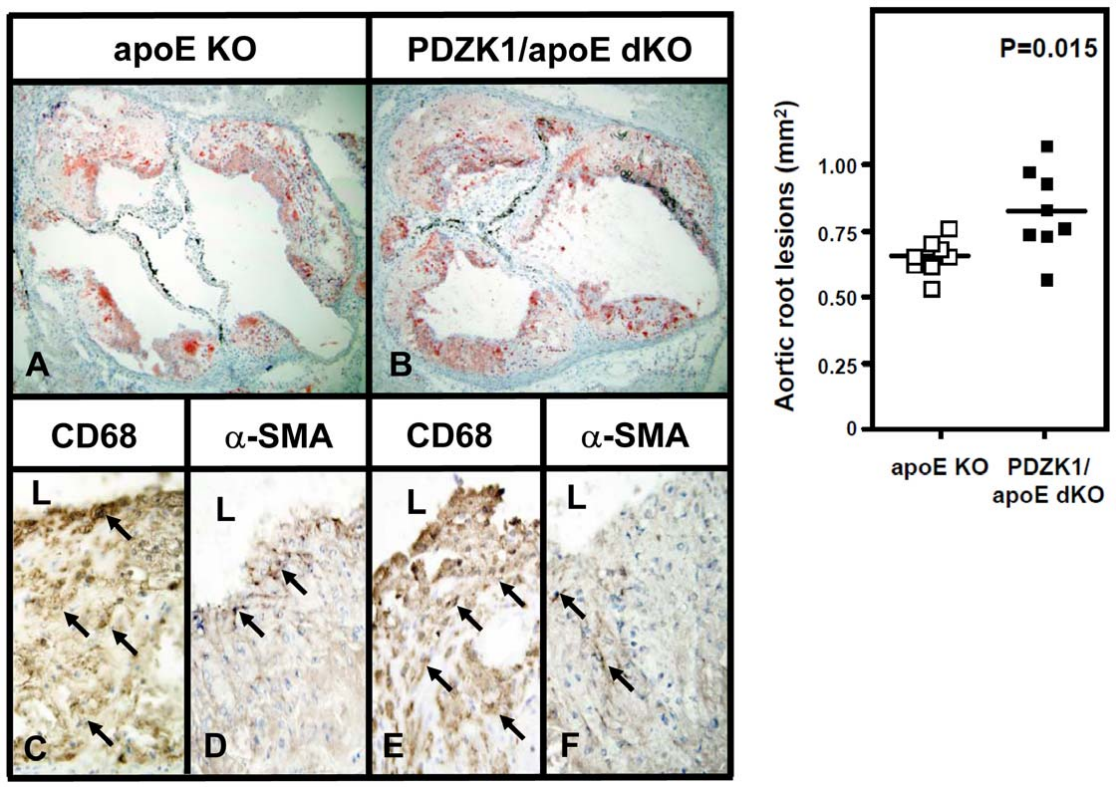
Aortic root atherosclerosis in Paigen diet-fed apoE KO and PDZK1/apoE dKO mice. Hearts were harvested from Paigen diet-fed apoE KO (A, C–D) and PDZK1/apoE dKO (B, E–F) mice as described in Methods (n = 8 per genotype). Left top panels: A–B: representative cross-sections of Oil red O-stained aortic root lesions. (magnification, ×20). Right top panel: quantification of aortic root atherosclerosis by planimetry. Unpaired Student's t-test was used to determine statistical significance. Bottom panels: immunohistochemistry of aortic root atherosclerotic plaques using CD68 (C and E) or alpha-smooth muscle actin α-SMA) (D and F) antibodies show that macrophages compose the overwhelming cell population of aortic root atherosclerotic plaques and that smooth muscle cells are rare in both apoE KO (C–D) and PDZK1/apoE dKO (E–F) mice. “L” indicates the vascular lumen, arrows indicate representative positive cells (magnification, ×100).

Hyperlipidemia is a risk factor for atherosclerosis and coronary heart disease [1]. Because of the increased plasma lipids in Paigen diet fed PDZK1/apoE dKO compared to apoE KO mice, we next evaluated atherosclerosis in the aortic roots and coronary arteries of these mice. Representative images of frozen sections of aortic roots, in which neutral lipids were stained with Oil Red O (top panels A and B), and quantification of this staining as a measure of atherosclerosis (right top panel) are shown in Figure 3. Both PDZK1/apoE dKO and apoE KO mice showed significant amounts of atherosclerosis. There was a 26% increase in the average lesion area in the aortic roots of PDZK1/apoE dKO mice compared to those of apoE KO mice (0.825 mm^2^±0.057 versus 0.654 mm^2^±0.024, 8 animals per group, P = 0.015). These results confirm our previous studies showing that PDZK1 is atheroprotective in the apoE KO mouse [21]. Immunohistochemical staining of the sections with antibodies that recognize macrophages (CD68) and smooth muscle cells (alpha-smooth muscle actin) showed that macrophages were overwhelmingly the most abundant cellular component of the atherosclerotic lesions in both apoE KO and PDZK1/apoE dKO mice, while smooth muscle cells were less abundant (Figure 3, C–F).

**Figure 4:**
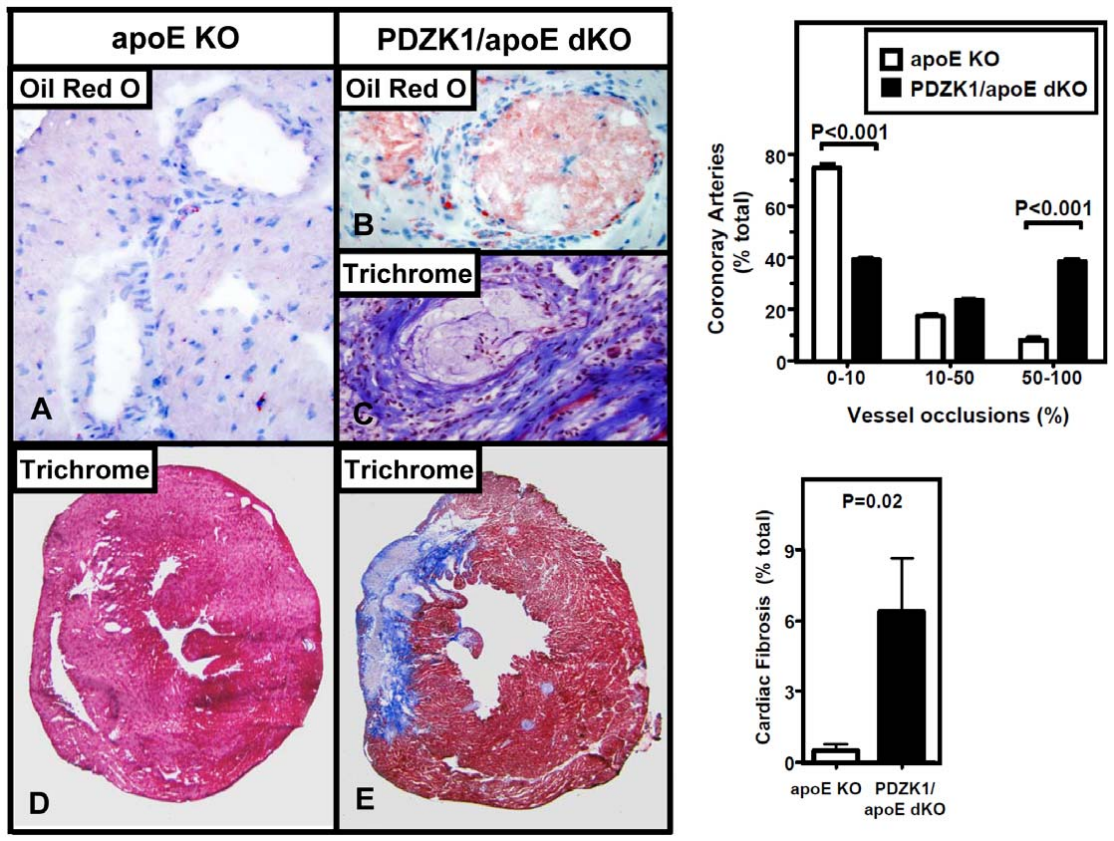
Effects of loss of PDZK1 on coronary atherosclerosis and cardiac fibrosis in apoE KO mice. Hearts were harvested from Paigen diet-fed mice as described in Methods (n = 8 per genotype). Left panels: A–B: representative cross-sections of Oil red O-stained (A–B) or trichrome-stained (C) myocardial coronary arterioles, showing unremarkable arterioles in apoE KO (A) and totally occluded arterioles in PDZK1/apoE dKO (B–C) mice (magnification, ×100). The Oil red O stain shows that the coronary arteriole is occluded almost exclusively by lipid-rich lesions (B), while the trichrome stain shows that the arteriole is surrounded by fibrosis in an area of myocardial infarction in a PDZK1/apoE dKO mouse (C). D–E: trichrome stained sections of hearts showing areas of infarction/fibrosis stained blue in PDZK1/apoE dKO (E), while they are absent in apoE KO (D) mice (magnification, ×10). Right top panel: quantification of coronary artery occlusions in apoE KO and PDZK1/apoE dKO mice. Statistically significant differences by ANOVA Tukey posthoc test comparing the two genotypes within a given group are indicated as: P<0.001. Right bottom panel: quantification of cardiac fibrosis in apoE KO and PDZK1/apoE dKO mice. Unpaired Student's t-test was used to determine statistical significance.

A common feature of human heart disease is occlusive coronary arterial atherosclerotic lesions and associated myocardial fibrosis. However, there are only a few murine models that robustly recapitulate these phenomena, especially after relatively short times (<6 months) of disease development [2], [11], [29], [42]. Notable examples include the SR-BI/apoE dKO mice on chow diet [11] and SR-BI/HypoE on a Paigen diet [42]. We previously did not observe substantial coronary arterial occlusion nor myocardial fibrosis in PDZK1/apoE dKO mice fed a Western diet for three months [21]. We therefore explored the effects of the Paigen-diet on these features of cardiac pathology. The extent of coronary arterial occlusions in apoE single KO and PDZK1/apoE dKO mice (Fig. 4, A–C) was assessed quantitatively by classifying the coronary arteries observed in frozen, stained cross sections into three categories depending on the severity of occlusions as follows: severely occluded (50–100% of the lumen occluded), partially occluded (10–50%) and minor occlusions (0–10%), as described previously [37], [38]. Occlusions were found in the mid- to distal portions of the coronary arteries. Oil Red O staining showed that these arteries were occluded almost exclusively by lipid-rich lesions, with very few cells present in the lesions (Fig. 4B). In addition, no thrombi were observed. Trichrome stained (Fig. 4C) images of occluded coronary arteries taken from the infarcted areas showed perivascular fibrosis, confirming that the infarcts were the direct consequence of the occluded coronary arteries. Figure 4 (right top panel) shows that in PDZK1/apoE dKO relative to apoE KO mice there was a 375% increase in the percentage of severely occluded (P<0.001) and with a concomitant 47% decrease in vessels with little or no occlusions (P<0.001).

Occluded coronary arteries are commonly implicated in myocardial infarctions in humans. To determine if the relatively high abundance of severely occluded arteries in PDZK1/apoE dKO mice were associated with higher incidence of myocardial infarction, we stained cardiac sections of both PDZK1/apoE dKO and apoE single KO mice with Masson trichrome to identify areas of myocardial infarction and fibrosis (blue stain, Fig. 4, D–E). ApoE single KO mice hearts showed no myocardial infarction and only occasional, minimal amounts of fibrosis, while myocardial infarctions were observed in 7 out of 8 (88%) PDZK1/apoE dKO mice. Quantitative evaluation of fibrosis indicated that there was ∼12 times more fibrosis in cardiac muscle of PDZK1/apoE dKO mice than in apoE KO mice (6.39%±2.2 vs 0. 52%±0.2, P = 0.02) (Fig. 4, right bottom panel). We did not observe evidence of gross cardiac dysfunction (cardiomagaly or pulmonary edema) in either experimental group. Indeed, no significant difference in cardiac size between apoE KO and PDZK1/apoE dKO mice was observed (e.g., heart weight to body weight ratios were 3.75±0.10 mg/g in apoE KO (n = 6) and 4.11±0.25 mg/g in PDZK1/apoE dKO (n = 8) mice, P = 0.27). A small percentage of mice died during the course of the study, however the death rate was not statistically different between the two genotypes (apoE KO: 4%, PDZK1/apoE dKO: 6.6%). The majority of deaths happened early in the course of the study (less that a month of feeding).

These results suggest that the PDZK1/apoE dKO mice subjected to Paigen diet represent a new animal model to study atherosclerotic coronary heart disease.

## Discussion

In this study, we describe a new murine model of diet-induced coronary heart disease, the Paigen-diet-fed PDZK1/apoE dKO mouse. ApoE KO and PDZK1/apoE dKO mice were fed a high fat/high cholesterol diet supplemented with cholate (Paigen diet [36]) for three months to study the consequences of PDZK1 gene inactivation on lipoprotein metabolism, atherosclerosis and coronary heart disease. Previously, we had shown that PDZK1 deficiency enhances aortic root atherosclerosis in apoE deficient mice on a Western-type high fat/high cholesterol diet, but did not markedly induce occlusive coronary arterial atherosclerosis or myocardial infarction [21]. Consistent with our previous results, we found that PDZK1 is atheroprotective for apoE mice fed the more severe Paigen diet. PDZK1/apoE dKO mice exhibited a 26% increase in the areas of aortic root lesions compared to apoE KO mice. However, unlike the case with Western diet feeding [21], we found that Paigen diet-fed PDZK1/apoE dKO, but not apoE single KO, mice were characterized by substantial occlusive coronary artery disease and the presence of myocardial infarctions.

The dramatic loss of hepatic SR-BI protein in the livers of Paigen diet-fed PDZK1/apoE KO mice clearly contributed to their dyslipidemia and possibly played a role to the relatively unusual murine early onset occlusive coronary artery disease. In addition, it is possible that the loss of PDZK1 in apoE KO mice might influence atherosclerosis and coronary heart disease via, as yet not identified, SR-BI-independent pathways.

Loss of PDZK1 in apoE KO mice resulted in elevated plasma unesterified cholesterol, UC:TC ratio and phospholipids on both Western diet- and Paigen diet-fed mice ([21], this study), but only in the Paigen diet-fed PDZK1/apoE dKO mice did we observe statistically significant elevations in total plasma cholesterol and phospholipids. The influence of these differences in plasma lipoproteins on the differences in occlusive coronary arterial disease in these mice remains to be determined. We previously showed that the inactivation of PDZK1 did not affect SR-BI levels in two cell types known to be implicated in development of atherosclerosis, macrophages and endothelial cells [21]. In addition, loss of PDZK1 did not affect the uptake of lipid from HDL in macrophages [21]. However, Shaul et al. have reported that SR-BI dependent regulation of endothelial cell function (e.g., control of eNOS activity by extracellular HDL) is disrupted in the absence of PDZK1 [43]. Thus, a disruption of SR-BI dependent endothelial cell function might have contributed to the diet-induced occlusive coronary artery disease.

Hyperlipidemia is a well known risk factor for coronary heart disease [1]. Complete loss of SR-BI in normal chow-fed apoE KO mice causes severe hypercholesterolemia associated with coronary arterial occlusion, myocardial infarction and premature death [11], whereas PDZK1/apoE dKO mice fed either chow or Western high fat diets do not exhibit cardiac fibrosis, myocardial infarction nor premature cardiac death [21]. In the present study, we show that feeding PDZK1/apoE dKO mice with the more severe Paigen diet resulted in the formation of coronary occlusions and cardiac fibrosis, but this was not associated with symptoms of cardiac dysfunction (e.g., cardiomegaly) during the 3 month duration of this study. The reason(s) for the less severe pathophysiology in PDZK1/apoE dKO compared to SR-BI/apoE dKO mice remain to be established. It might be attributed to the atheroprotective effect of residual SR-BI expression that persists following inactivation of the PDZK1 gene (5% in the liver, 50% in the small intestine and unchanged in steroidogenic organs, endothelial cells and macrophages) [21], [32]. In addition, the level of hepatic expression of a minor SR-BI isoform, SR-BII [44], is unaffected by the loss of PDZK1 [32]. One distinctive difference in lipoprotein metabolism between chow-fed PDZK1/apoE and SR-BI/apoE dKO mice that is likely to reflect differences in the tissue specificity and/or absolute levels of SR-BI/II expression and function is the large difference in the UC:TC ratios in their plasma lipoproteins: ∼0.8 in SR-BI/apoE dKO [45] and ∼0.4 in PDZK1/apoE dKO [21] mice (∼0.25 in wild-type mice). However, the relatively low absolute values and small relative differences for the plasma UC:TC ratios in Paigen diet-fed apoE KO (0.27) and PDZK1/apoE dKO (0.31) mice caste doubt on the importance of this characteristic of plasma lipoproteins in influencing coronary arterial atherogenesis and myocardial infarction in this system.

The PDZK1/apoE dKO mouse model joins a growing list of animal models that share features of classic human coronary heart disease [11], [29], [42], [46] and may thus prove useful for characterizing mechanisms underlying disease development and testing approaches for prevention and treatment. Our findings also suggest that PDZK1 itself might be an attractive target for pharmacologic intervention for targeted therapies.
